# iPSC villages to understand neuroimmune changes in Alzheimer's disease

**DOI:** 10.1002/alz70855_103942

**Published:** 2025-12-24

**Authors:** Martine Therrien

**Affiliations:** ^1^ University of California Davis, Davis, CA, USA

## Abstract

**Background:**

Genome‐wide association studies have expanded our understanding of late‐onset Alzheimer's disease (LOAD) genetics and identified multiple loci increasing the risk for disease, including in APOE and TREM2. Still, only a fraction of the heritability can be explained by those variants, and new studies suggest complex genetic architectures, such as polygenic or oligogenic contributions to LOAD. To recreate these complex interactions, we need new models to understand the impact of genetic risk on disease progression.

Multiple risk genes are expressed in myeloid cells, including microglia. Recently, we have shown that iPSC‐derived microglia (iMG) differentiated in vitro and responding to specific brain challenges can adapt complex transcriptional signatures similar to those observed in the brain. This project aims to test microglia village to study the impact of LOAD genetics across hundreds of patient iMG.

**Method:**

To scale up iMG culture, we adapted a pooled culture technique called villages. Cells from multiple donors are pooled early in the differentiation, and the phenotype is linked to each donor using variants specific to each individual. This method has been used to identify modifiers of neuronal toxicity but microglia pose some intrinsic challenges.

**Result:**

Using village for iMG in vitro and xenograft models, we show that microglia from up to 50 different donors grown in villages recapitulate microglia states observed in vitro and in vivo. We found that donors are equally distributed across multiple transcriptional signatures, including disease‐associated microglia transcriptional signatures. This method also allows us to study microglia function across multiple donors. For example, we confirmed the defects in phagocytosis in TREM2R47H carrying donors. Finally, villages can be used to identify new variants altering key microglia characteristics. In a pool of 25 cell lines, we identified TMEM106B, a lysosomal protein involved in neurodegenerative disease, as a modifier of microglia phagocytosis

**Conclusion:**

Microglia villages can be used to study the impact of LOAD accurately and uncover phenotypes across multiple patients, including males and females and patients from various genetic ancestry. This method will allow us to bridge the gap between transcriptomic signature and microglia function to identify regulators of microglia states and functions across a large number of patients.

